# Clinical laboratory evaluation of the Orion SS-20 ionized calcium analyser

**DOI:** 10.1155/S1463924679000298

**Published:** 1979

**Authors:** Patrick Ferreira, Alan M. Bold

**Affiliations:** 1 Department ofLaboratory Medicine University of Alberta Hospital Edmonton Alberta T6G 2B7 Canada; 2 Department of Clinical Chemistry Queen Elizabeth Medical Centre Edgbaston Birmingham B15 2TH UK


for the gas stream in the distillation unit.

Coagulation of the beer samples, especially when running a large number of samples, can arise in the distillation unit. Coagulation interferes with the smooth flow of sample through the distillation unit and gives erratic results. Coagulation was prevented by mixing the sample with a 1 M potassium hydroxide solution (Figure 2) before pumping it into the distillation unit. The pH of the beer sample then varied between 6 and 7.

ACKNOWLEDGEMENTS

We are obliged to Technicon Scandinavia AB for all the help with the AutoAnalyzer II system and to Ing Thelemann (Warby Bryggeriet) for the beer samples.

REFERENCES

[1] West, D. B., Lautenbach, A. L. and Becker, K. *Proceedings of the American Society of Brewing Chemists,* 1952, 81.

[2] Kielhöfer, E. and Würdig, G. *Weinwissenschaft,* 1960, **15,** 135.

[3] Canales, A. M. and Martinez, N. *American Brewery,* 1962, 10.

[4] Brenner, M. W., Blick, S. R., Frenkel, G. and Siehenberg, J. *Proceedings of the European Brewery Convention,* Brussels, 1963, 233.

[5] Owades, J. L. and Jakovac, J. A. *Proceedings of the American Society of Brewing Chemists,* 1963, 22.

[6] Drews, B., Specht, H., Bärwald, G. and Trenel, G. *Monatsschrift Brauerei,* 1966, **19,** 34.

[7] Gjertsen, P., Undstrup, S. and Trolle, B. *Monatsschrift Brauerei,* 1964, **17,** 232.

[8] Harrison, G. A. *European Brewery Convention, Proceedings of the 10th Congress,* Stockholm, 1965.

[9] Arkim, A. *82nd Congress of the American Brewmasters in Toronto,* 1968.

[10] Bärwald, G. *Monatsschrift Brauerei,* 1970, **23,** 12.

[11] Buijten, J. *Blut Alkohol,* 1975, **12,** 393.

[12] Kaiser, R. E. and Rieder, R. *Chromatographia,* 1977, **10,** 455.

[13] Wainwright, T. *Journal of the Institute of Brewing,* London 1973, **79,** 451.

### Clinical laboratory evaluation of the Orion SS-20 ionized calcium analyser

**Patrick Ferreira**

*Department of Laboratory Medicine, University of Alberta Hospital, Edmonton, Alberta T6G 2B7, Canada.*

**Alan M. Bold**

*Department of Clinical Chemistry, Queen Elizabeth Medical Centre, Edgbaston, Birmingham B15 2TH, England.*

### Introduction

About 50% of the total plasma calcium exists as free calcium ions (ionized calcium, $Ca^{2+}$), and this is generally considered to be the physiologically active fraction. There is evidence to suggest that its direct measurement is of greater value than total calcium in various disorders [1,2]. Until recently techniques for measurement of ionised calcium (spectrophotometric [3-5] and potentiometric [6-12]) have been too unreliable or time-consuming for routine use in most laboratories. pH and temperature affect the binding of calcium to protein [4], and the necessity to control these variables has led to further analytical complications.

With the introduction of the Orion model SS-20 ('Space Stat'-20) this situation could change. A recent advertisement [13] for the analyser stated: 'Operation is simple: the sample is injected, a button is pushed and the result read on the digital display. Now this important blood parameter can be measured throughout the hospital — the central laboratory, in satellite laboratories, in intensive care, in pediatrics. Ionized calcium can now join the list of routine clinical tests.'

Indeed, five recent publications have essentially supported this claim [14-18], but the authors' experience has not been so favourable and some shortcomings of the instrument have been found in this evaluation that have not been previously recognized. These findings are factors which should be considered by potential users of the instrument.

### Materials and methods

### The SS-20 analyser

It is not intended to give a detailed description of the analyser, this is available from the manufacturer: Orion Research Inc., 380 Putnam Ave., Cambridge, Mass. 02139, U.S.A. The instrument is basically designed to measure $Ca^{2+}$ at 37°C in an anaerobic sample of whole blood, plasma or serum. This is performed in a three minute automated cycle in which the sample is pumped past a liquid ion-exchange type of calcium electrode (the 'sensor'). The reference electrode is Ag/AgCl through which a slow flow of 2 mol/l KCl saturated with $Ag^+$ is pumped, this then meets the sample stream at a liquid junction. A standard (1.00 mmol/l $Ca^{2+}$) is pumped past the sensor following the sample. From the difference in potentials, and using a 'slope' factor calibrated in each run using aqueous standards, the instrument automatically calculates and displays the $Ca^{2+}$ result. The reagents and standards used were as those supplied by the manufacturer, and the analyser was used in accordance with the manufacturer's instruction manual. Sensor potentials were monitored on a chart recorder throughout the study.

### Ionized calcium: spectrophotometric method

The method of Varghese [5] was used: This involved preliminary anaerobic ultrafiltration at 37°C, followed by dual-wave-length spectrophotometry of tetramethyl murexide added to the ultrafiltrate.

### Dialysable calcium method

The method of Ferreira and Bold [19] was used. This involves continuous-flow dialysis at 37°C of an anaerobic sample, with detection of calcium in the dialysate using o-cresolphthalein complexone.

pH was measured using a Radiometer BMS Mk2 blood gas analyser.

Total calcium was measured by the standard SMA-12/60 o-cresolphthalein complexone method (based on No. $AA_203$). (Technicon Corp., Tarrytown, U.S.A.).

### Sample preparation

Venous blood was collected into heparinized polypropylene syringes, which were capped. The dilution of heparin was chosen to give a final concentration of 3-4 units/ml of whole blood. The syringes were centrifuged, nozzles uppermost, and the separated plasma was drawn off into a second set of syringes using short sleeves of plastic tubing to connect the syringe nozzles. All analyses were performed within four hours of collection.

### Control sera

Three levels of $Ca^{2+}$ were prepared by pooling hospital in-patient samples (excluding jaundiced or haemolysed sera). The 'high'

control was unmodified, as the $Ca^{2+}$ of the pool was slightly elevated. The 'medium' control was prepared by diluting the pool 4:1 v/v with 140 mmol/l NaCl. The 'low' control was prepared by dialysis in cellulose tubing ('Visking' supplied by R. W. Jennings & Co., Nottingham, U.K.) against a solution containing 140 mmol/l NaCl, and 0.8 mmol/l $MgCl_2$, but no calcium.

Each control was aliquotted and stored frozen at —20°C. Aliquots were equilibrated with 6.3% $CO_2$ before analysis, using an IL237 Tonometer. (Instrumentation Laboratory, Lexington, U.S.A.).

### Results and discussion

### Precision

Results are summarised in Table I. In the three control sera, the day-to-day range of pH found was 7.37-7.42 for the high control, 7.30-7.34 in the medium control, and 7.23-7.28 in the low control.

*Figure 1 Quality Control Chart: Day-to-day results on control sera and normal subjects have been plotted on 10 days over a period of six weeks. The SS-20 results (bold lines) are very variable, despite correct slope adjustment and standardization in every run using aqueous standards. For comparison, the faint lines show concurrent spectrophotometric $Ca^{++}$ and dialysable calcium results; these do not show similar day-to-day variations.*

*Key:*
——— *SS-20 $Ca^{2+}$ results*
--------- *Spectrophotometric $Ca^{2+}$ results*
— — — *Dialysable calcium results*
● *High Control*
◆ *Medium Control*
▲ *Low Control*
○ *Mean of 3 normals*
□ *One individual*

The only previous investigation of day-to-day precision of the SS-20 using serum samples [16] demonstrated worse precision than within-day: day-to-day coefficient of variation (CV) ranged from 1.4-3.9 times the corresponding within-day values (the results obtained from this study were 1.7-2.8 times within-day). In addition a considerably better day-to-day precision was reported using aqueous solution. The authors' results show similar large differences between within-day and day-to-day precision, but a worse overall precision. The within-day precision data obtained agreed with that reported by Fuchs et al, [14]; however theseauthors found no difference in precision using whole blood or plasma samples, whereas the whole blood measurements in this study are considerably less precise.

### Variable response with sensor age

The reason for the poor day-to-day precision is apparent from Figure 1: the sensor apparently varies from day-to-day in its response to serum samples, relative to its response to aqueous standards. These variations are statistically significant: the mean apparent $Ca^{2+}$ of four normal subjects on day 4 was lower than day 2 ($p < 0.01$), then fell further on day 5 ($p < 0.01$) and rose again on day 6 ($p < 0.001$), coincident with sensor replacement. (p values calculated using Student's unpaired t-test). Similar patterns have been observed on other occasions using different sensors; generally results on serum or plasma samples fall (relative to aqueous standards) with advancing sensor age. (Rotation of the sensor (Figure 1) probably 'rejuvenates' it by exposing a fresh area of membrane to the sample).

It was also observed that this variable sensor response did not occur with dialysed serum. A serum pool was dialysed in Visking tubing against a large volume of dialysate containing 140 mmol/l NaCl, 5 mmol/l KCl and 0.7 mmol/l $MgCl_2$; adjusted to pH 7.4 with conc. HCl and aliquots of different $Ca^{2+}$ (approx. 0.5-2.0 mmol/l) were prepared by the addition of appropriate volumes of 100 mmol/l $CaCl_2$. Results were consistent day-to-day: the dialysed sera gave responses similar to aqueous standards, whereas undialysed serum and plasma samples run concurrently

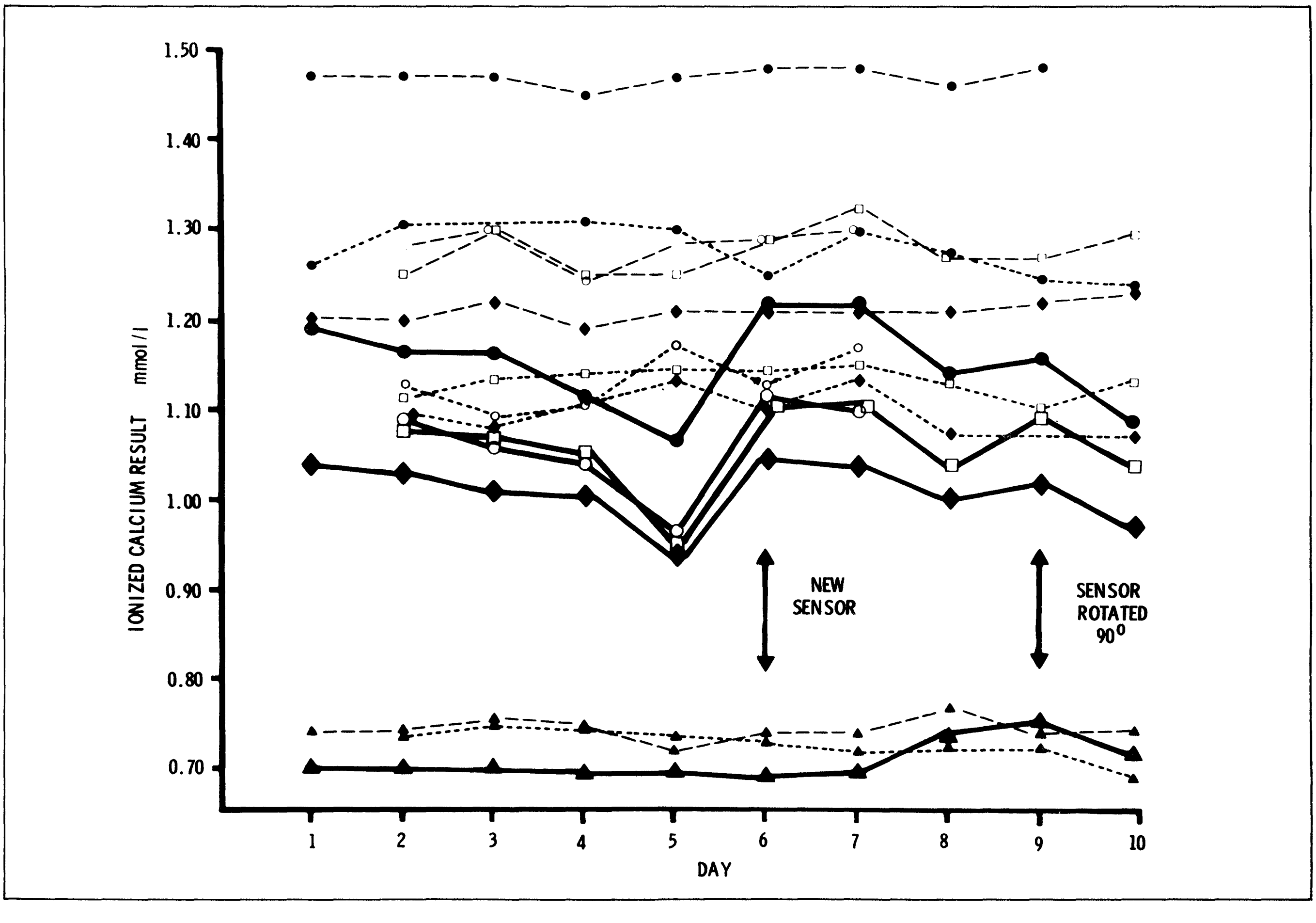

**Table I Precision Data**

| | Sample | n | Ionized Calcium (mmol/l) mean | s.d. | c.v. % |
|---|---|---|---|---|---|
| 1. *SS-20 Analyser* | | | | | |
| *a) Within-day** | High Control | 10 | 1.16 | 0.017 | 1.5 |
| | Medium Control | 11 | 1.01 | 0.013 | 1.3 |
| | Low Control | 11 | 0.70 | 0.015 | 2.1 |
| 15 normal subjects: | Plasma | 15 | 1.09 | 0.017 | 1.5 |
| (Group B) | Whole Blood | 15 | 1.27 | 0.036 | 2.8 |
| b) *Day-to-Day* | High Control | 9 | 1.16 | 0.049 | 4.2 |
| | Medium Control | 9 | 1.02 | 0.032 | 3.1 |
| | Low Control | 9 | 0.71 | 0.026 | 3.6 |
| 2. *Spectrophotometric Method* | | | | | |
| a) *Within-day** | High Control | 8 | 1.28 | 0.023 | 1.8 |
| | Medium Control | 7 | 1.10 | 0.010 | 0.9 |
| | Low Control | 8 | 0.73 | 0.024 | 3.3 |
| | Normal Subjects | 26 | 1.06 | 0.021 | 2.0 |
| b) *Day-to-day* | High Control | 8 | 1.28 | 0.026 | 2.1 |
| | Medium Control | 7 | 1.10 | 0.028 | 2.5 |
| | Low Control | 8 | 0.73 | 0.010 | 1.3 |

* Calculated from n duplicates.

**Table II Normal Reference Values**

| 1. *Ionized Calcium (mmol/l)* *Spectrophotometric method* | | n | mean | s.d. | mean 2±s.d. | c.v. % |
|---|---|---|---|---|---|---|
| | Group A | 19 | 1.13 | 0.031 | 1.071-1.195 | 2.7 |
| | One Individual | 9 | 1.13 | 0.016 | 1.100-1.164 | 1.4 |
| *SS-20 Analyser* | | | | | | |
| Group A: | Results Uncorrected | 20 | 1.07 | 0.046 | 0.975-1.160 | 4.3 |
| | Results Corrected* | 20 | 1.11 | 0.023 | 1.065-1.159 | 2.1 |
| Group B: | Whole Blood | 15 | 1.27 | 0.036 | 1.198-1.341 | 2.8 |
| (All analysed in one run) | Plasma | 15 | 1.09 | 0.024 | 1.042-1.138 | 2.2 |
| One Individual: | | | | | | |
| | Results Uncorrected | 9 | 1.06 | 0.049 | 0.960-1.158 | 4.7 |
| | Results Corrected* | 9 | 1.11 | 0.029 | 1.052-1.169 | 2.6 |
| 2. *Total Calcium (mmol/l)* | | | | | | |
| | Group A | 20 | 2.41 | 0.074 | 2.259-2.554 | 3.1 |
| | One Individual | 9 | 2.36 | 0.064 | 2.229-2.484 | 2.7 |
| 3. *Hydrogen Ion Concentration ([H+] nmol/l)* | | | | | | |
| Group A: | Whole Blood | 20 | 46.0 | 1.67 | 42.6-49.3 | 3.6 |
| | Plasma | 20 | 42.8 | 1.66 | 39.5-46.2 | 3.9 |
| Group B: | Whole Blood | 15 | 45.9 | 3.51 | 38.9-52.9 | 7.7 |
| | Plasma | 15 | 41.5 | 3.00 | 35.5-47.5 | 7.2 |
| One Individual: | Whole Blood | 9 | 44.6 | 2.30 | 40.0-49.1 | 5.2 |
| | Plasma | 9 | 42.0 | 1.58 | 38.8-45.2 | 3.8 |

* Method of 'correction' of results is explained in text under 'serum standardization'.

showed significant day-to-day variability. The low control serum used in this study which had been dialysed did not show as much day-to-day variability as the other controls.

**Serum standardization**

To minimize day-to-day errors due to the phenomenon described above, a 'serum standardization' procedure was tried and evaluated. Since there is no reference method for $Ca^{2+}$ determination, results on control sera given by a new sensor on day 6 were arbitrarily chosen as being 'correct' and results on other days were related to them (Figure 2), forming a series of 'calibration lines'. From these lines, results on other samples could be 'corrected' to their expected values on day 6.

This resulted in more consistent day-to-day results (Figure 3), also evidenced by (a) the narrowing of a normal reference interval that was established over several days (Group A, Table II), to a similar range that was established on a single day (Group B, Table II); (b) by the narrowing of apparent intraindividual variation (Table II); and (c) by improved correlation with the spectrophotometric method. (Schwartz has also advocated the use of serum standards with the 'Electrion' system [11]).

**Reference values (Table II)**

Group A comprised 20 healthy, ambulant laboratory staff (10 male, 10 female) aged 18-50 years. Specimens were collected and analysed on 7 separate days over the course of 5 weeks. One individual from the group was sampled on 9 days over 5 weeks.

Group B comprised 15 healthy, ambulant university students (8 male, 7 female) aged 18-22 years. All samples were collected and analysed on a single day. Both groups were non-fasting, collected mid-morning. None of the distributions differed significantly from Gaussian (Kolmogorov-Smirnov test, $p > 0.05$). No sex difference was observed (Mann-Witney test, $p > 0.05$).

Some of the apparent difference in $[H^+]$ between whole blood and plasma is probably on artefact, since red cells effect the liquid junction of the pH electrode system [20,21].

The wide uncorrected normal reference values agree quite well with those quoted by Orion (SS-20 instruction manual) and Madsen and Olgard [16], through Hudsan et al, [17] found somewhat narrower limits. However the corrected reference range reported here is the narrowest so far published. This would imply that the wider limits found by other workers could be at least

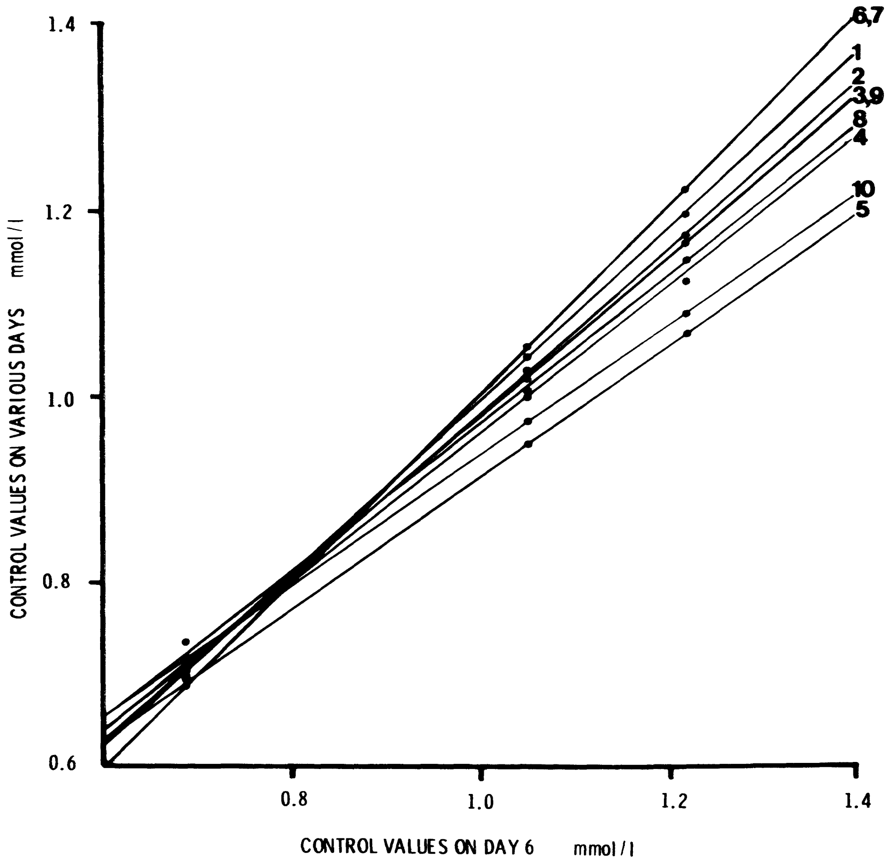

*Figure 2 Calibration lines for "correction" of SS-20 results.* The control serum results on each day have been plotted against those on day 6, when a new sensor was fitted. Results of samples on each day can thus be corrected to their expected values on day 6 by reading off the appropriate line.

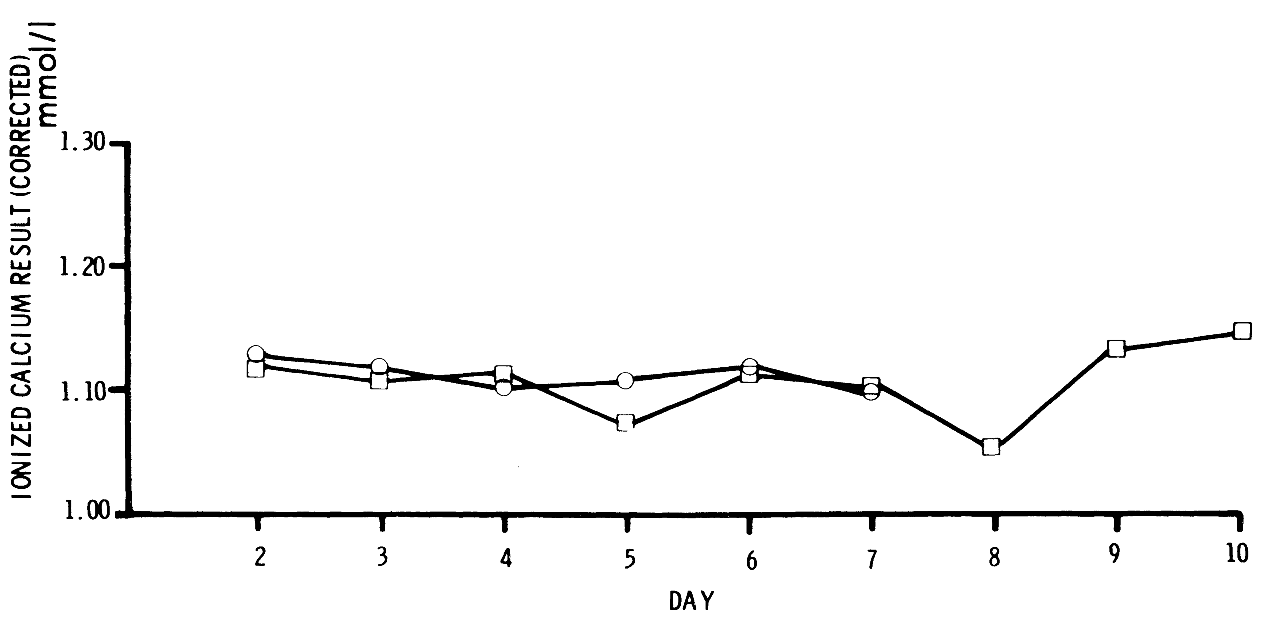

*Figure 3 SS-20 Results after "correction."* Day-to-day variation in results is considerably diminished after applying corrections based on control serum values. (Compare Figure 1)

O Mean of 3 normal subjects

□ One individual.

partly explained by the same day-to-day variability in sensor response that is described here.

### Comparison with the spectrophotometric method

There was good correlation between the methods (Figure 4); the SS-20 results were a mean of 0.021 mmol/l lower (significant, Wilcoxon signed rank test, $p < 0.001$). If uncorrected SS-20 results were used the correlation coefficient between the methods was worse, at 0.935.

### Interferences

***Red cells.*** Results on whole blood were a mean of 0.18 mmol/l higher than on plasma samples (Table II, Group B). The difference is highly significant (Wilcoxson signed rank test, $p < 0.001$). (The small difference in $[H^+]$ would only account for approximately 0.025 mmol/l difference in $Ca^{2+}$ [4,12]). The effect is dependent on the haematocrit (Figure 5). Both Madsen and Olgard [16], and Larsson and Öhman [22] have found that results are higher using whole blood, through neither report so large a difference as reported in this work. Fuchs et al [14] found no difference. There is also a report of higher results on whole blood using a previous Orion electrode [8].

The phenomenon may be explained by changes in the residual liquid junction potential caused by red cells [20,22,23]; also possibly the fact that pH of whole blood is less likely to change in the SS-20 tubing (see below under 'pH stability').

### Protein

The effect of protein on the SS-20 was tested by preparing samples with varying protein content, but with essentially the same $Ca^{2+}$. Each control serum was separated into three fractions using anaerobic ultra-filtration at 37°C [5]. The protein-free ultrafiltrate, the native serum, and the protein-rich retentate were then analysed in quadruplicate by the SS-20. The $Ca^{2+}$ in each fraction would be expected to be similar since cation retention during ultrafiltration (the Donnan effect) would be roughly counter-balanced by protein retention, causing relative volume change [3,4]. Dialysable calcium measurements supported this expectation, being similar in all fractions. However the SS-20 results suggested positive interference by protein; the mean results, expressed as a percentage of the ultrafiltrate value (i.e. 100%) were: native serum 108%, and protein-rich retentate 117%. This is approximately +0.0135 mmol/l apparent $Ca^{2+}$ per 10 g/l increase in protein concentration.

That this might be a general effect of colloids was suggested in a further experiment where 75 g/l of Dextran 70 (mol. wt. 70,000, Pharmacia, Sweden) was incorporated in a 1.00 mmol/l $CaCl_2$ solution (in a matrix of 140 mmol/l NaCl, 3 mmol/l tris/HCl, pH 7.0). The apparent $Ca^{2+}$ measured in triplicate by the SS-20 was 1.07 mmol/l, but the dialysable calcium result was unchanged at 1.00 mmol/l.

This effect may be explained on the basis of changes in the residual liquid junction potential, and also by volume exclusion effects of colloids. It has not been reported previously. The 'interference' noted by Madsen and Olgard [16] of albumin added to blood, serum or aqueous standard, is simply a reflection of the calcium binding properties of albumin, and not a direct effect of albumin on the analytical method.

### Heparin

Other authors have noted that heparin lowers $Ca^{2+}$ because of

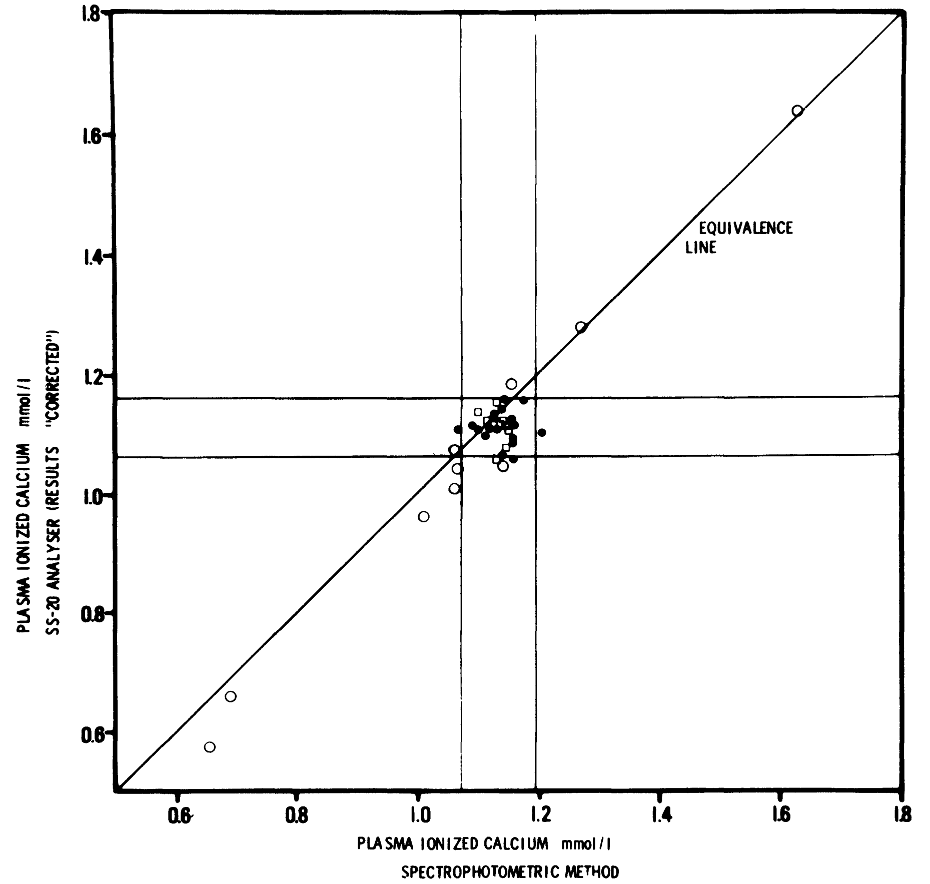

*Figure 4 Comparison of two ionized calcium methods.* Regression equation: Y = 1.04x—0.07; r = 0.973 n = 37.

- ● Normal individuals (Group A). Reference intervals indicated.
- □ One individual
- ○ Patients with miscellaneous disorders affecting $Ca^{2+}$ levels.

complex formation [10,17] and possibly changes in the residual liquid junction potential [22]. The authors results support the former, since changes in dialysable calcium parallel those of $Ca^{2+}$ (Figure 6). At the concentration used in this study the effect is less than 2%, in agreement with Madsen and Olgard [16].

**Miscellaneous interference**

Interference from $Na^+$, $K^+$, $Mg^{++}$, $H^+$ and Tris buffer was assessed by incorporating each in 1.00 mmol/l $CaCl_2$ in at least 4 different concentrations. Interference was calculated from regression coefficients of apparent $Ca^{2+}$ against concentration of interfering substance (Table III). (The correlation coefficients were significant ($p < 0.001$) except in the case of $K^+$).

*Sodium Effect:* A slight negative interference found for sodium conflicts with the report of a marked positive effect [16]. Previous Orion electrodes have been noted to show either positive [10] or negligible [6] sodium error.

*Magnesium Effect:* Results found here are similar to those of Madsen and Olgard [16] but results using previous Orion electrodes were confusing, both positive [6] and negative [7,10] errors being noted.

*pH Effect:* There has not been a previous study of the effect of pH on SS-20 results. (The experiment of Madsen and Olgard [16] altering the pH of serum simply demonstrates the pH effect of calcium binding to protein, not direct analytical interference). The effect found is somewhat similar to that reported with a previous Orion electrode [9].

Both pH and magnesium interference appear to arise by altering the time course of the sensor response (Figure 7). It is certainly advisable to use buffered standards: there is a slight constant bias introduced by using 3 mmol/l Tris/HCl whereas unknown pH changes could cause serious and variable errors. It might be preferable to use standards buffered at pH 7.4 (we found Orion standards to be approximately pH 7.0), and containing 0.7 mmol/l $MgCl_2$ to more closely simulate the composition of normal plasma.

**Table III Miscellaneous Interferences**

| | *Concentration Range* | *Interference** |
|---|---|---|
| $Na^+(Cl^-)$ | 100-170 mmol/l | —0.00046 |
| $Mg^{++}(2Cl^-)$ | 0-3 mmol/l | +0.0210 |
| Tris/HCl(pH 7.0) | 0-12 mmol/l | —0.0047 |
| pH** | 6.81-7.98 | —0.0058 |
| $K^+(Cl^-)$ | 0-40 mmol/l | No significant effect |

* expressed as mmol/l apparent $Ca^{2+}$ per 1.00 mmol/l increase in the interferring substance (0.1 unit increase in the case of pH).

** buffered with 3.0 mmol/l Tris/HCl (pH is used since interference is linear with pH, rather than $[H^+]$).

All solutions contained 1.00 mmol/l $CaCl_2$ and (except the varying $Na^+$ solutions), 140 mmol/l NaCl.

### pH stability

The SS-20 is designed to convey samples anaerobically to the sensor in order to avoid pH rise due to loss of $CO_2$ that would in turn cause increased calcium binding to protein, and consequent lowering of $Ca^{2+}$. It was found that anaerobically collected whole blood rose in pH by 0.03 units and plasma by 0.06 units after 30 seconds in the silicone rubber tubing used in the SS-20 analyser,

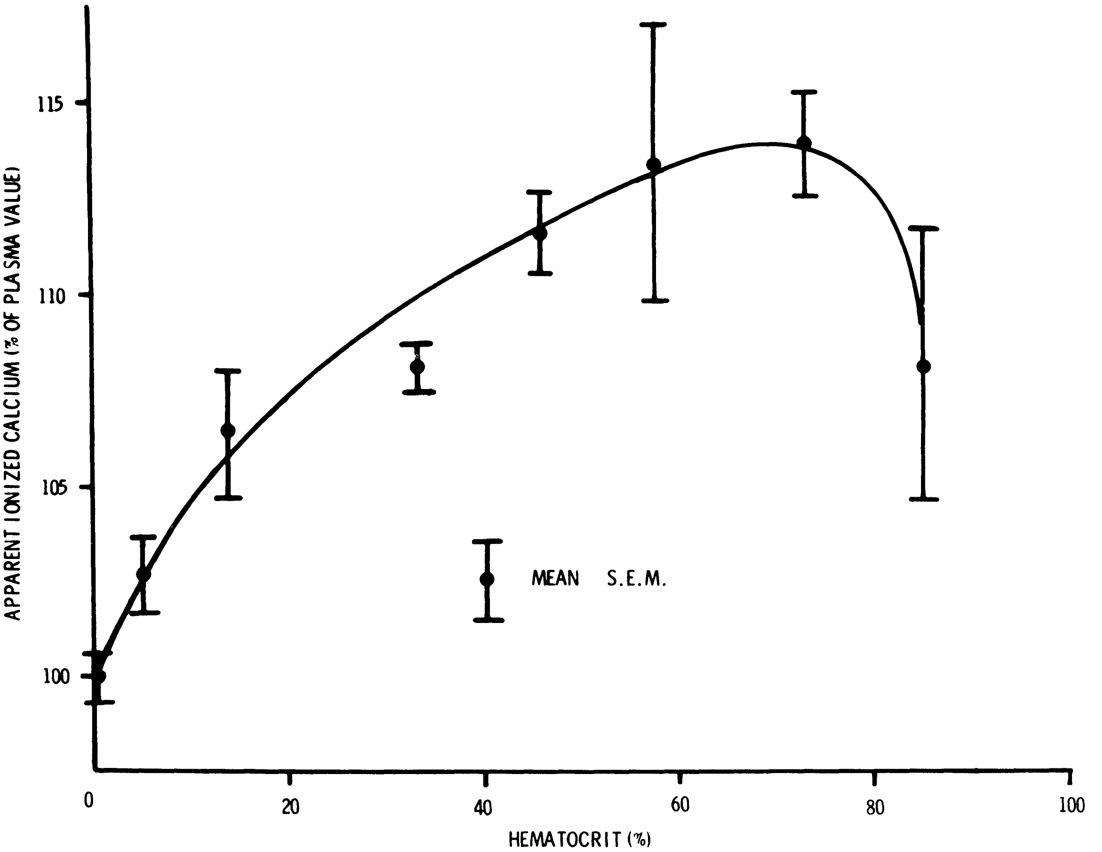

*Figure 5 Effect of hematocrit on SS-20 results.* Samples of varying hematocrit but the same $Ca^{2+}$ were prepared *in vitro* by anaerobically recombining cells and plasma from one original whole blood specimen. Pooled results of four such experiments are shown.

but there was no change in pH when the experiment was repeated using polypropylene tubing of similar dimensions. This has a significant effect on results (Table IV), and is probably due to the fact that silicone rubber is permeable to $CO_2$ (in fact it has been used for specifically that purpose, in membranes of $CO_2$ electrodes [24]).

The smaller pH change in whole blood is probably because of its greater buffering capacity; this could also partly explain why results on whole blood are higher than plasma, since the pH of plasma would tend to rise more rapidly. Indeed the difference becomes less when polypropylene tubing is substituted. The variability of the reported difference in results between whole blood and plasma, both in this study and others [14,16,22] could be due to variation in the time the sample is exposed to the SS-20 tubing before analysis; i.e. variations in the speed of sample injection and how promptly the analyser is started. The true extent of pH change in the tubing may have been underestimated in this experiment, since the rise in $Ca^{2+}$ values after fitting polypropylene tubing was out of proportion to the observed improvement in pH stability. (Approximately 0.05 mmol/l increase in $Ca^{2+}$ is expected per 0.1 pH decrease [4,12]).

**Linearity**

There is some deviation from linearity at low $Ca^{2+}$ (Figure 8). Examinations of chart recordings of sensor potentials showed that non-linearity could be ascribed to the sensor, not the electronic signal processing.

'Recovery' of calcium added to or removed from serum was also linear (Figure 9).

### Error in first sample

The value measured for the first sample analysed tended to be falsely low by as much as 15%. The error was greatest if more than 10 minutes had elapsed since the last sample, being about 2% low at 5 minutes, with no detectable error at 2 minutes. It was noted using both aqueous standards and plasma samples; from chart recordings it appeared to be due to a slow sensor response.

**Table IV pH stability of a sample in the SS-20 analyser**

| | *SS-20 Tubing* (silicone rubber)* | | *Polypropylene Tubing** | |
|---|---|---|---|---|
| | *pH change*** | *SS-20 result (mmol/l)* | *pH change*** | *SS-20 result (mmol/l)* |
| Whole Blood*** | +0.04 | 1.142 | +0.02 | 1.237 |
| Plasma*** | +0.11 | 1.075 | +0.03 | 1.187 |

* Refers to removable tubing between the injection port and sensor, namely the sample inlet tubing and holding coil.

** Refers to change in pH measured between samples collected (using blood gas capillary collection tubes) from the injection syringe and the 'overflow outlet' of the analyser during an analytical cycle. (i.e. The rise in pH after transversing the analyser tubing for approximately 45 seconds).

*** A single sample was collected from a normal subject, and part of it anaerobically separated. All $Ca^{2+}$ measurements were in triplicate.

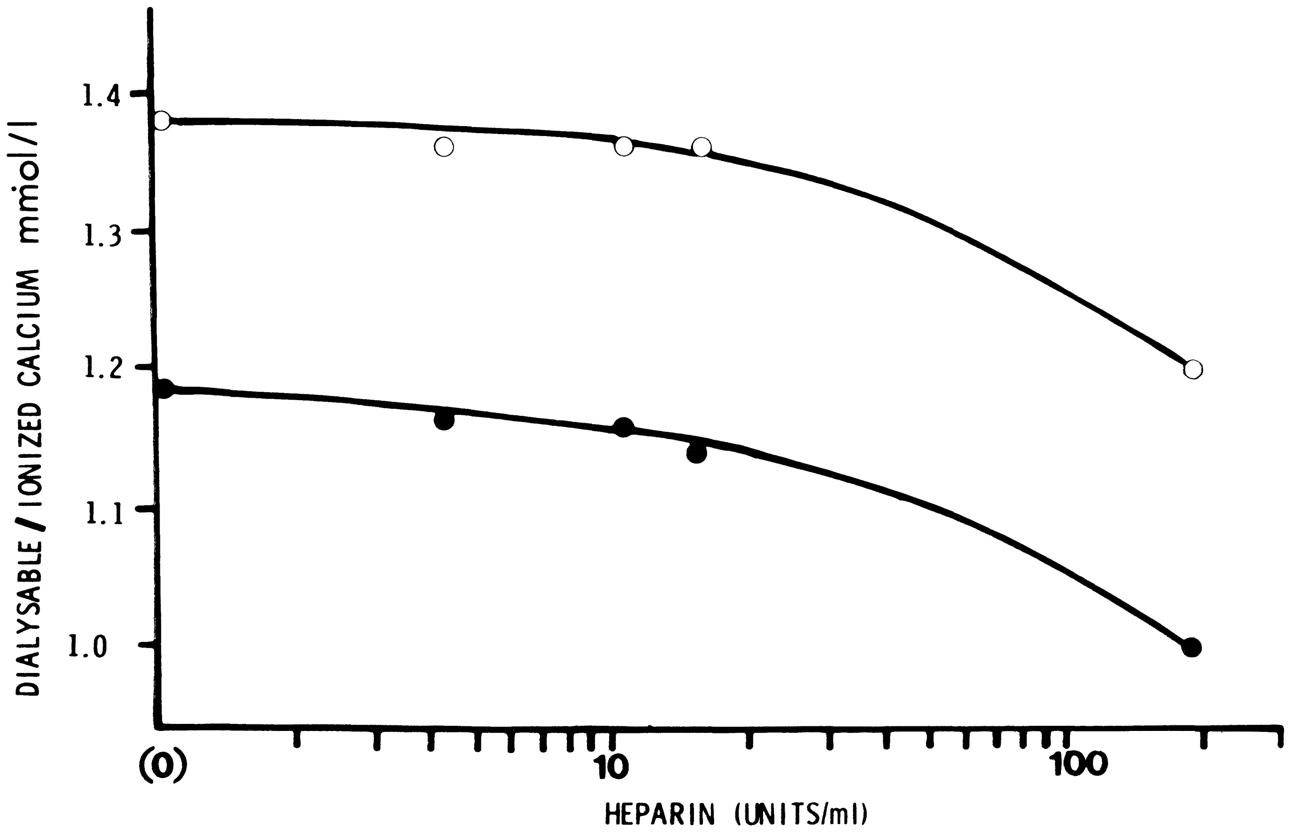

*Figure 6 Effect of heparin.* Heparin was added to the high control serum in various concentrations.

O Dialysable calcium results

● SS-20 $Ca^{2+}$ results.

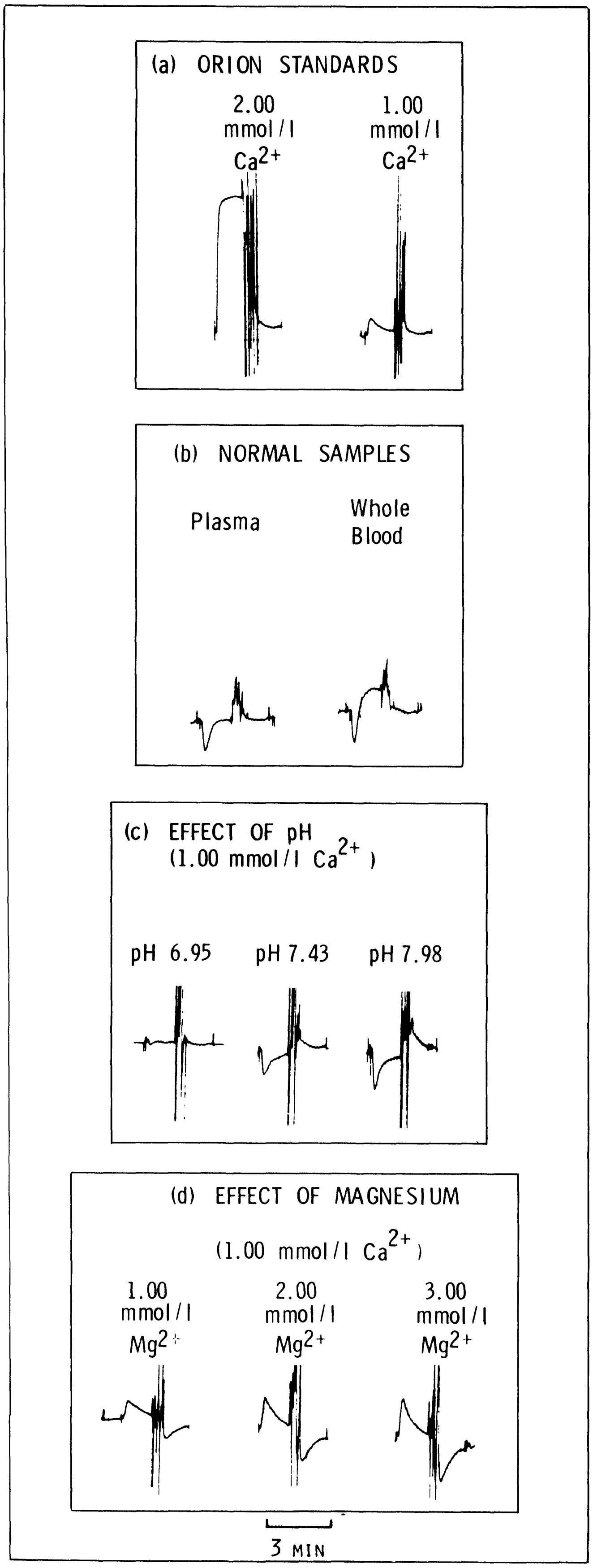

*Figure 7 Chart-recordings of sensor potentials.* To the left in each tracing is the sample measurement period, to the right is the internal standard measurement period, with air/standard wash in between. Increasing pH and increasing $Mg^{++}$ appear to delay the attainment of 'plateau' potentials, both in sample and standard measurement periods.

This error tended to diminish with advancing sensor age.

Dependability was good: No major problems occurred in 6 months use. Most frequently, any malfunction noted was due to blockage or kinking of tubing. The use of a chart recorder was a valuable adjunct in detecting malfunction, and in trouble shooting.

### Conclusions

1. There appears to be a dialysable factor in plasma that gives rise to day-to-day variability in results, despite correct aqueous standardization. It is essential to run day-to-day control sera to detect such inconsistancies in sensor response. Serum standardization appears to be useful in correcting for such errors.
2. Whole blood samples are not recommended since precision is worse, and results are artefactually high to an extent dependent on the hematocrit, and possibly other factors.
3. In view of different protein, pH, $Mg^{++}$, $Na^{+}$, Tris and heparin content between plasma samples and Orion aqueous standards, we would endorse the opinion of Larsson and Ohman [20] that results cannot be taken as representing absolute measurements of $Ca^{2+}$ activity mainly because of the uncertain and variable residual liquid junction potential.
4. A change of sample tubing and holding coil to a material impermeable to $CO_2$ is recommended.
5. Samples should be run in continuous batches, no more than 2 minutes separating each analytical cycle, and ignoring the first result of the batch.
6. An accurate analysis is not as simple as is implied in advertisements [13]. Reliable results can only be obtained by a skilled operator who has a thorough knowledge of the possible pitfalls involved. With this proviso however, the SS-20 analyser is certainly an advance over previous systems and will undoubtedly be a valuable tool in many clinical laboratories.

A direct comparison with the alternative "Electrion" system [11] would be of great interest.

ACKNOWLEDGEMENTS
The SS-20 Analyser was purchased with a grant from the Central Birmingham Health District Medical Research Endowment Fund.

REFERENCES
[1] Robertson, W. G. *Ann. Clin. Biochem.*, 1976, **13**, 540.
[2] Ladenson, J. H. and Bowers, G. N. *Clin Chem.*, 1973, **19**, 575.
[3] Walser, M. *J. Clin. Invest.*, 1961, **40**, 723.
[4] Pederson, K. O. *Scand. J. Clin. Lab. Invest.*, 1970, **25**, 223.

*Figure 8 Linearity of response of SS-20 using agueous standards.* (All standard solutions contained calcium, as chloride, in NaCl 140 mmol/l and tris/HCl 3 mmol/l, pH 7.0).

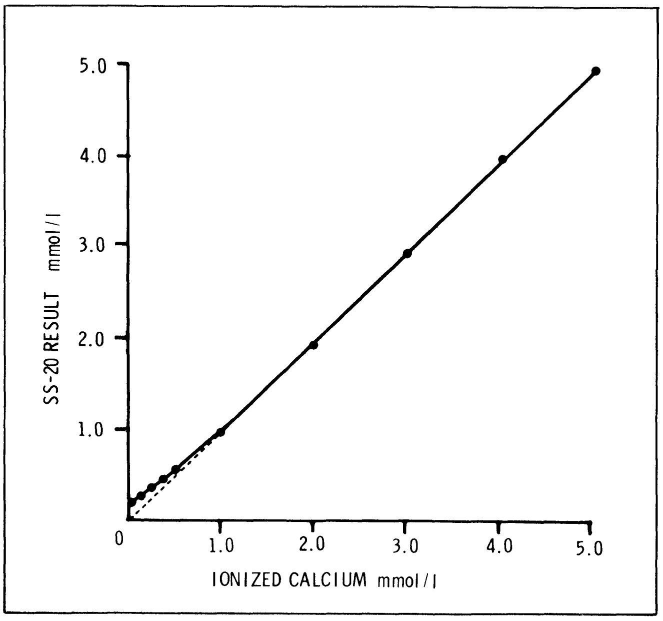

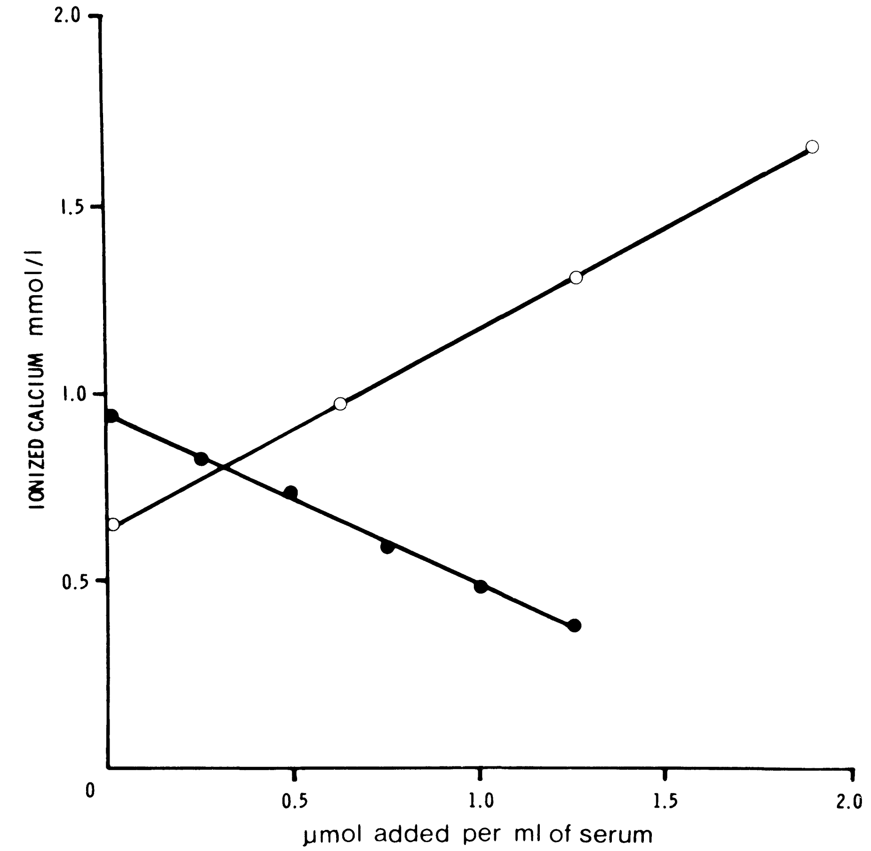

*Figure 9 Recovery experiments.* Calcium was added (o) or removed (•) from serum by the addition of various concentrations of $CaCl_2$ and EDTA, respectively.

[5] Varghese, Z. *Ann. Clin. Biochem.*, 1973, **10**, 120.

[6] Moore, E. M. In: Ion selective electrodes, 1969, (Durst, R. A., Ed) p.215, Nat. Bur. Stand. (U.S.) Spec. Publ. 314.

[7] Li, T. F. and Piechocki, J. T. *Clin. Chem.*, 1971, **17**, 411.

[8] Sacks, L. L., Bordeau, A. M. and Presle, V. *Rev. Eur. Étud. Clin. Biol.*, 1971, **16**, 831.

[9] Rushton, M. L., Sammons, H. G., Gosling, P. and Robinson, B. H. B. *Ann. Clin. Biochem.*, 1973, **10**, 63.

[10] Ladenson, J. H. and Bowers, G. N. *Clin. Chem.*, 1973, **19**, 565.

[11] Schwartz, H. D. *Clin. Chim. Acta.*, 1975, **64**, 227.

[12] Wybenga, D. R., Ibbott, F. A. and Cannon, D. C. *Clin. Chem.*, 1976, **22**, 1009.

[13] Orion Research Inc.: Advertisement *Clin. Chem.*, 1977, **23**, (9) 38A.

[14] Fuchs, C., Dorn, D., McIntosh, C. and Scheler, F. *Clin. Chim. Acta.*, 1976, **67**, 99.

[15] Alpert, N. L. *Lab. World.*, 1976, **27**, 40.

[16] Madsen, S. and Ølgard, K. *Clin. Chem.*, 1977, **23**, 690.

[17] Husdan, H., Leung, M., Oreopoulos, D. and Rapoport, A. *Clin. Chem.*, 1977, **23**, 1775.

[18] Conceicao, S. C., Ward, M. K., Alvarez-Ude, F., Aljama, P., Smith, P. and Kerr, D. N. S. *Clin. Chim. Acta.*, 1978, **86**, 143.

[19] Ferreira, P. and Bold, A. M. Abstract 181, *Xth Int. Cong. Clin. Chem.*, 1978, Mexico.

[20] Severinghaus, J. W., Stupfel, M. and Bradley, A. F. *J. Appl. Physiol.*, 1956, **9**, 189.

[21] Blayo, M. C., Helfgott, P. and Pocidalo, J. J. *Rev. Fr. Étude. Clin. Biol.*, 1963, **8**, 1021.

[22] Larsson, L. and Öhman, S. *Clin. Chem.*, 1978, **24**, 731.

[23] Khuri, R. N. In: Ion selective electrodes, 1969, (Durst, R. A. Ed.) p.287, Nat. Bur. Stand. (U.S.) Spec. Publ. 314.

[24] Sternberg, J. C., Updike, S. J. and Lehane, D. P. in: Microtechniques for the Clinical Laboratory, 1976, (Werner, M. Ed.) p.142.

